# Adverse events and nocebo phenomena: treatment or disease specific?

**DOI:** 10.1186/s12916-019-1272-2

**Published:** 2019-02-05

**Authors:** Panagiotis Zis, Panagiota Sykioti

**Affiliations:** 1 Medical School, University of Cyprus, Nicosia, Cyprus; 2 Academic Department of Neurosciences, Sheffield Teaching Hospitals NHS Foundation Trust, Sheffield, UK; 3 Collingham Child and Family Centre, Central and North West London NHS Foundation Trust, London, UK

**Keywords:** Nocebo, adverse events, trial design

## Background

The quality of recording and reporting of adverse events (AEs) in randomized clinical trials (RCTs) has, in the past, been less rigorous than that of the recording and reporting of efficacy [1]. However, such data has been systematically provided in recent years. In their recent article in *BMC Medicine* article, Bolton et al. [2], using a meta-analytic approach, examined serious adverse events (SAEs) and AEs occurring in clinical trials of oral naltrexone administered for various conditions compared to placebo. Meta-analyses of this kind are vital in the clinical context since they provide information on the safety and tolerability of a specific intervention. Bolton et al. [2] concluded that naltrexone does not appear to increase the risk of SAEs over placebo, whereas it increases the risk of certain AEs of mild nature (i.e., decreased appetite, dizziness, nausea). Correctly, the authors further assessed whether the prevalence of AEs and SAEs fluctuated across the various disease groups, finding no significant differences. Thus, this brings to attention the question on whether AEs are treatment or disease specific.

### Lessons from the nocebo meta-analyses

In the current literature, safety and tolerability meta-analyses focus on analyzing RCTs of various interventions in a single disease, analyzing RCTs of a single intervention in one disease, or studying RCTs of a single intervention in more than one disease. A minority of the available meta-analyses focuses on nocebo phenomena, defined as AEs occurring in patients receiving a placebo treatment. Establishing the extent of nocebo phenomena allows us to estimate, indirectly, what percentage of the AEs reported to occur in the active treatment arms of RCTs is actually a result of the negative expectations of patients that a treatment will harm, rather than heal.

Nocebo meta-analyses have been conducted in neurological or psychiatric diseases pooling data from RCTs of various treatments in single diseases [3–7]. Nocebo AEs and dropout rates vary significantly and depend on the pathophysiology of the given disease [8]; indeed, diseases involving the central nervous system tend to have higher nocebo AE rates and subsequently higher active treatment-related AEs [3]. This is in agreement with Zaccara et al. [9], who showed that nocebo rates in RCTs are affected by the clinical condition for which the experimental drug is given. For example, placebo-treated patients with obesity and binge eating disorders and those with pain have significantly higher proportions of intolerable AEs requiring drug withdrawal compared to placebo-treated epilepsy patients on anticonvulsants.

### Should we be focusing on SAEs, AEs, or both?

Although Bolton et al. [2] primarily focused on the prevalence of SAEs, which is a method to assess an intervention’s safety, they also performed a secondary analysis of AEs as a measure of an intervention’s tolerability. This is a very important methodological aspect in such meta-analyses, since even non-serious AEs can lead to discontinuation of treatment, which was indeed shown to happen in the case of naltrexone.

### The importance of AE reporting

How RCT results are reported may minimize concerns and detection of AEs. Even in RCTs published in high-impact journals, explicit mention of AEs might be missing, reducing the quality of the study [10]. Bolton et al. [2] have used the Cochrane Risk of Bias Tool for quality assessment, thus reducing the risk of including RCTs of poor quality in their meta-analysis.

### Methodological limitations

Nevertheless, the results of the Bolton et al. [2] meta-analysis should be interpreted with caution, given that the included populations across the RCTs were not homogenous; it included RCTs where naltrexone was tested in different diseases. However, to a certain degree, this limitation is overruled by the fact that the authors investigated whether the figures differed across the various diseases. Additionally, as occurs in all meta-analyses of this type, it is of particular importance to highlight that patients might have several comorbidities for which they receive other treatments, which may also lead to AEs. Although the majority of RCTs require adherence to other treatments for specific time periods, possible interactions between the new drug and those already being administered cannot be adequately studied and evaluated.

Another important limitation, also common in meta-analyses of this type, is that despite AEs being often classified as drug-related in RCTs, the inherent difficulty in attributing non-specific symptoms is a potential source of bias. Similarly, it can be difficult to distinguish whether symptoms documented as AEs arise subsequent to drug administration or are a consequence of the natural history of a disease.

## Conclusion

The key finding in Bolton et al.’s study [2] is that naltrexone is a safe medication. However, although it is generally well tolerated, it carries an increased risk (relative risk 1.33) of treatment discontinuation because of its AEs compared to placebo.

AEs are both treatment and disease specific; for example, an anticonvulsant might show a slightly different AE profile and frequency when used for the treatment of neuropathic pain than when used for the treatment of epilepsy. Therefore, meta-analyses of RCTs testing a single treatment in a single disease, when possible, are more likely to provide clinically useful information. However, when the number of the available single-intervention trials in a single disease is small, and single-intervention RCTs in multiple diseases are pooled together, further analyses should be performed to elucidate differences across the various diseases.
